# Clinicopathological characteristics, prognostic factors, and outcomes of elderly patients with lymphoma‐associated hemophagocytic lymphohistiocytosis: A multicenter analysis

**DOI:** 10.1002/cam4.70178

**Published:** 2024-09-01

**Authors:** Yi Miao, Jing Zhang, Xuzhang Lu, Meng Wu, Bingzong Li, Liang Yu, Miao Sun, Yun Zhuang, Yuqing Miao, Haiwen Ni, Xiaoyan Xie, Jingyan Xu, Yunping Zhang, Min Zhao, Min Xu, Wanchuan Zhuang, Weiying Gu, Guoqiang Lin, Haiying Hua, Jianfeng Zhu, Maozhong Xu, Tao Jia, Ping Liu, Lijia Zhai, Tongtong Zhang, Qiurong Shan, Qiudan Shen, Jun Qian, Chunling Wang, Jianyong Li, Wenyu Shi

**Affiliations:** ^1^ Department of Hematology The First Affiliated Hospital of Nanjing Medical University Nanjing China; ^2^ Jiangsu Cooperative Lymphoma Group (JCLG) and Jiangsu Histiocytosis Association Lymphoma Group Nanjing China; ^3^ Department of Hematology Affiliated Changzhou Second Hospital of Nanjing Medical University Changzhou China; ^4^ Department of Oncology Affiliated Hospital of Nantong University Nantong China; ^5^ Department of Hematology The Second Affiliated Hospital of Soochow University Suzhou China; ^6^ Department of Hematology The First People's Hospital of Huai'an Huai'an China; ^7^ Department of Hematology, Jingjiang People's Hospital The Seventh Affiliated Hospital of Yangzhou University Jingjiang China; ^8^ Department of Hematology Wuxi People's Hospital Wuxi China; ^9^ Department of Hematology Yancheng First People's Hospital Yancheng China; ^10^ Department of Hematology The Affiliated Hospital of Nanjing University of Traditional Chinese Medicine Nanjing China; ^11^ Department of Hematology Northern Jiangsu People's Hospital Affiliated to Yangzhou University Yangzhou China; ^12^ Department of Hematology, Nanjing Drum Tower Hospital The Affiliated Hospital of Nanjing University Medical School Nanjing China; ^13^ Department of Hematology The Affiliated Yixing Hospital of Jiangsu University Yixing China; ^14^ Department of Hematology Wuhu Second People's Hospital Wuhu China; ^15^ Department of Hematology Zhangjiagang First Affiliated Hospital of Soochow University Zhangjiagang China; ^16^ Department of Hematology The Second People's Hospital of Lianyungang Lianyungang China; ^17^ Department of Hematology The First People's Hospital of Changzhou and The Third Affiliated Hospital of Soochow University Changzhou China; ^18^ Department of Hematology Huai'an Hospital Affiliated to Xuzhou Medical College and Huai'an Second People's Hospital Huai'an China; ^19^ Department of Hematology Affiliated Hospital of Jiangnan University Wuxi China; ^20^ Department of Hematology The People's Hospital of Taizhou Taizhou China; ^21^ Department of Hematology The Affiliated Jiangyin Hospital of Southeast University Medical College Jiangyin China; ^22^ Department of Hematology The First People's Hospital of Lianyungang Lianyungang China; ^23^ Department of Hematology Wuxi Second People's Hospital Wuxi China; ^24^ Department of Hematology Affiliated Hospital of Yangzhou University Yangzhou China; ^25^ Department of Hematology Rudong County People's Hospital Rudong China; ^26^ Department of Hematology Shuyang Traditional Chinese Medicine Hospital Shuyang China; ^27^ Department of Hematology The Affiliated Suzhou Hospital of Nanjing Medical University, Suzhou Municipal Hospital Suzhou China; ^28^ Department of Hematology Affiliated People's Hospital of Jiangsu University Zhenjiang China

**Keywords:** elderly patients, hemophagocytic lymphohistiocytosis, lymphoma, overall survival

## Abstract

**Background:**

Lymphoma is the most common secondary cause of hemophagocytic lymphohistiocytosis (HLH) in adults. Lymphoma‐associated HLH (LA‐HLH) in the elderly population is not rare, however, little has been reported regarding clinicopathological characteristics, prognostic factors, and outcomes of LA‐HLH in the elderly population.

**Methods:**

We retrospectively analyzed a multicenter cohort of elderly patients with LA‐HLH. Clinicopathological features and treatment information were collected. The impacts of baseline characteristics and treatments on survival outcomes were analyzed.

**Results:**

A total of 173 elderly patients with LA‐HLH were included. Compared with young patients, elderly patients showed different clinical and laboratory features. Regarding lymphoma subtypes, B‐cell lymphoma was more common in elderly patients (elderly 61.3% vs. young 32.3%, *p* < 0.001) while T/NK‐cell lymphoma was more common in young patients (65.3% vs. 35.3%, *p* < 0.001). The median survival of elderly patients with LA‐HLH was only 92 days. The prior use of HLH therapy or etoposide‐containing HLH therapy was not associated with improved overall survival. T/NK‐cell subtype, a lower platelet count (≤53 × 10^9^/L), a lower albumin level (≤32.1 g/L), a higher LDH level (>1407 U/L), and a higher creatinine level (>96.8 μmol/L) were independent predictors of decreased overall survival and 60‐day survival. A prognostic index was established and demonstrated to be robust in predicting the overall survival and 60‐day survival of elderly patients with LA‐HLH.

**Conclusions:**

LA‐HLH in elderly patients displayed heterogeneous clinicopathological features and survival outcomes. Treatments need to be optimized to improve the outcomes of elderly patients with LA‐HLH.

## 1 INTRODUCTION

Hemophagocytic lymphohistiocytosis (HLH) is a rare clinical syndrome characterized by overwhelmed inflammation. HLH could be classified as primary HLH and secondary HLH. Primary HLH is caused by germline genetic defects and is always present in infants and children.^1^ Secondary HLH has underlying causes and is typically identified in adults. The common underlying causes of secondary HLH include malignancies, infections, and rheumatic disorders. In the context of malignancies, lymphoma is the most common type of malignancy, accounting for over 70% of cases.^2^ Geographical variation in lymphoma subtypes exists in patients with lymphoma‐associated HLH (LA‐HLH). In China, T/NK‐cell lymphoma is more common than B‐cell lymphoma as a trigger for adult HLH and NK cell malignancies are the major triggers.^3^, ^4^, ^5^ By contrast, in Europe and USA, large B cell lymphoma and T‐cell non‐Hodgkin lymphoma are the main underlying causes of LA‐HLH and a significant proportion of cases of LA‐HLH were Hodgkin lymphomas.^6^, ^7^ The lymphoma subtypes have significant impacts on the prognosis of LA‐HLH with T/NK‐cell lymphoma predicting significantly worse outcomes.^6^ The current recommendation for treating LA‐HLH is controlling hyperinflammation by HLH‐directed regimens, followed by lymphoma‐directed therapy.^8^

The majority of cases of LA‐HLH are adult cases. Whether patients in different age groups show distinct clinicopathological features and outcomes remains to be determined. Little is known about LA‐HLH in the elderly population. As elderly patients have more comorbidities and reduced tolerance to chemotherapy, elderly patients with LA‐HLH may need a different therapeutic approach. Therefore, we retrospectively analyzed the largest cohort focusing on elderly patients with LA‐HLH to date, to explore clinical features, lymphoma subtypes, prognostic features, therapy, and outcomes of LA‐HLH in the elderly population.

## 2 METHODS

### 2.1 Study population

This study was a multicenter, retrospective study focusing on elderly patients with LA‐HLH. Data on young patients were also collected for comparison purposes. Adult patients with LA‐HLH diagnosed at the participating centers from 2010 to 2023 were included in this study. The inclusion criteria were as follows: (1) age ≥18 years; (2) fulfillment of five or more of the eight HLH‐2004 diagnostic criteria^9^; and (3) histopathological diagnosis of lymphoma. Primary HLH caused by germline genetic abnormalities was excluded. The lymphoma subtyping was based on the World Health Organization classification of lymphoid neoplasms. Patients >60 years were defined as elderly patients. Young patients (18 years ≤ age ≤60 years) were included for comparison with their elderly counterparts.

### 2.2 Data collection

The demographic data, clinical features, and laboratory results of all patients were extracted from the patient's medical records. Demographic data were age at diagnosis and gender. Clinical characteristics include the duration of fever and the maximum temperature, the presence of splenomegaly, and the lymphoma stage at diagnosis. The subtypes of the underlying lymphoid malignancies were also collected. Laboratory parameters included hemoglobin, platelets, absolute neutrophil count, soluble CD25, ferritin, triglycerides, fibrinogen, natural killer (NK) cell activity, lactate dehydrogenase (LDH), albumin, and creatinine. The values of laboratory parameters for diagnosis of HLH and prognosis analysis were documented separately. For the parameters in the HLH‐2004 diagnostic criteria, the first values that meet the HLH‐2004 criteria were obtained. For parameters included in prognosis analysis, the latest laboratory values measured before a specific HLH‐directed or lymphoma‐directed therapy were used. When no data were available, the first value within 72 h after treatment initiation was documented. For patients who did not receive any HLH‐directed or lymphoma‐directed therapy, baseline laboratory parameters at the time of diagnosis of HLH were used for prognosis analysis.

### 2.3 Statistical analyses

The Mann–Whitney test was used to compare the continuous variables between the two groups. Fisher's exact test or chi‐square test was used for categorical variables. The survival curves were plotted by using the Kaplan–Meier method and the log‐rank test was used for comparison. In prognostic analysis, we used the R package “survminer” to determine the optimal cutoffs for continuous variables including age, hemoglobin, platelets, neutrophils, albumin, creatinine, LDH, and fibrinogen. We used 1500 μg/L as the cutoff for ferritin as there were no concrete values in some cases with high ferritin levels (for example, ferritin>1500 μg/L). Univariate and multivariate Cox regression analyses were performed to find the predictors of overall survival (OS) and 60‐day survival. Statistical analyses were performed using STATA/MP, version 17.0 (StataCorp LLC) and R Software, version 4.2.2. *P* < 0.05 was considered statistically significant.

## 3 RESULTS

### 3.1 General data and clinical features

A total of 173 elderly patients with LA‐HLH were included. A separate cohort of 251 young patients with LA‐HLH was used for comparison studies. The median age was 69 years, and the male/female ratio was approximately 1.8:1 (Table 1). Almost all the cases were in advanced stages (99.4%). No HLH presented during chemotherapy was identified in the elderly patients. Regarding the HLH diagnostic parameters, 92.9% (158/170) of patients showed persistent fever, and 87.3% (151/173) of patients had splenomegaly; Cytopenia affecting ≥ two lineages were present in 90.8% of patients; hypertriglyceridemia and hypofibrinogenemia were identified in 37.0% (64/173) and 55.5% (96/173) of cases, respectively; hemophagocytosis in bone marrow was present in 80.6% (133/165) of cases; 98.3% (169/172) and 94.8% (109/115) of cases showed hyperferritinemia and elevated soluble CD25; only 27 patients were evaluated for NK cell activity with 51.9% showing reduced NK cell activity. Persistent fever was less frequent in elderly patients. Absent or reduced NK cell activity was less frequent in elderly patients as compared with young patients, although the difference was not statistically significant. Elderly patients show significantly decreased albumin levels and increased creatinine levels, suggesting that organ functions were decreased in elderly patients.

**TABLE 1:** Demographic, clinical, and laboratory characteristics of elderly and young patients with lymphoma‐associated hemophagocytic lymphohistiocytosis.

	Elderly patients	Young patients	*p* Value
	(*N* = 173)	(*N* = 251)	
Age at diagnosis, median (IQR), years	69 (65–74)	47 (35–55)	<0.001
Male	111/173 (64.2)	166/251 (66.1)	0.675
Fever ≥38.5°C	158/170 (92.9)	241/248 (97.2)	0.055
Splenomegaly	151/173 (87.3)	223/251 (88.8)	0.624
Cytopenia (≥ 2 of 3 lineages)	157/173 (90.8)	224/251 (89.2)	0.613
Triacylglycerol ≥3 mmol/L	64/173 (37.0)	103/249 (41.4)	0.366
Fibrinogen ≤1.5 g/L	96/173 (55.5)	128/251 (51.0)	0.362
Hypertriglyceridemia and/or hypofibrinogenemia	124/173 (71.7)	179/251 (71.3)	0.935
Hemophagocytosis	133/165 (80.6)	195/239 (81.6)	0.804
Ferritin ≥500 μg/L	169/172 (98.3)	242/251 (96.4)	0.375
Ferritin ≥1500 μg/L	115/172 (66.9)	160/250 (64.0)	0.544
Elevated soluble CD25^a^	109/115 (94.8)	164/181 (90.6)	0.191
Low NK cell activity	14/27 (51.9)	22/30 (73.3)	0.093
Advanced stages	160/161 (99.4)	232/237 (97.9)	0.408
Albumin, median (IQR), g/L	26.95 (24.2–31.7)	28.8 (25.4–32.5)	0.009
Creatinine, median (IQR), umol/L	62.05 (52.2–80.3)	56.7 (44.8–70.8)	<0.001
Lactate dehydrogenase (IQR), U/L	572.5 (352.5–1214.5)	635.5 (369–1251.5)	0.890

*Note*: Data are given as number/number of cases with available data (%) unless stated otherwise.

Abbreviation: IQR, interquartile range.

^a^ Elevated soluble CD25 was defined as soluble CD25 >2400 U/mL or soluble CD25 >6400 pg/mL.

### 3.2 Lymphoma subtypes in elderly patients with lymphoma‐associated HLH

In elderly patients with LA‐HLH, 106 patients had B‐cell non‐Hodgkin lymphoma (NHL), 61 patients had T/NK‐cell lymphoma, and six patients had Hodgkin lymphoma. The most common subtype was large B‐cell lymphoma (56.7%, 98/173), followed by angioimmunoblastic T‐cell lymphoma (AITL, 9.3%, 16/173), aggressive NK cell leukemia (ANKL, 6.4%, 11/173), extranodal NK cell lymphoma, nasal type (ENKL, 5.8%, 10/173), and peripheral T‐cell lymphoma, not otherwise specified (PTCL‐NOS, 5.8%, 10/173). It is important to note that the proportion of B‐cell NHL was significantly higher in elderly patients than in young patients (elderly 61.3% vs. young 32.3%, *p* < 0.001). And the T/NK‐cell lymphoma was less common in elderly patients (35.3% vs. 65.3%, *p* < 0.001). Furthermore, we compared the proportions of specific subtypes between elderly and young patients. Large B‐cell lymphoma was more common in elderly patients, while ANKL and ENKL were more common in younger patients than in older patients. AITL was more frequent in older patients, although the difference was only marginally significant (Table 2).

**TABLE 2:** Lymphoma subtypes in elderly and patients with lymphoma‐associated hemophagocytic lymphohistiocytosis.

Subtypes	Elderly patients	Young patients	*p* Value
	*N* (%)	*N* (%)	
Overall	173	251	
B‐cell non‐Hodgkin lymphoma	106 (61.3)	81 (32.3)	<0.001
Large B‐cell lymphoma^a^	98 (56.7)	74 (29.5)	<0.001
B‐NHL	5 (2.9)	4 (1.6)	0.496
Burkitt lymphoma	2 (1.2)	0	0.166
CLL	1 (0.6)	1 (0.4)	1.000
MZL	0	1 (0.4)	1.000
WM	0	1 (0.4)	1.000
T/NK‐cell lymphoma	61 (35.3)	164 (65.3)	<0.001
AITL	16 (9.3)	12 (4.8)	0.069
ANKL	11 (6.4)	49 (19.5)	<0.001
ENKL	10 (5.8)	49 (19.5)	<0.001
PTCL, NOS	10 (5.8)	19 (7.6)	0.473
T cell lymphoma, NOS	6 (3.5)	25 (10.0)	0.012
EBV‐associated T/NK‐LPD	3 (1.7)	8 (3.2)	0.537
ALCL	2 (1.2)	1 (0.4)	0.570
Gamma‐delta T cell lymphoma	2 (1.2)	1 (0.4)	0.570
T‐LGLL	1 (0.6)	0	0.408
Hodgkin lymphoma	6 (3.5)	5 (2.0)	0.368
Composite	0	1 (0.4)	1.000

Abbreviations: AITL, angioimmunoblastic T‐cell lymphoma; ALCL, anaplastic T‐cell lymphoma; ANKL, aggressive NK cell leukemia; B‐NHL, B‐cell non‐Hodgkin lymphoma; CLL, chronic lymphocytic leukemia; EBV‐associated T/NK‐LPD, EBV‐associated T/NK‐cell lymphoproliferative diseases; ENKL, extranodal NK/T cell lymphoma, nasal type; MZL, marginal zone lymphoma; PTCL, NOS, peripheral T‐cell lymphoma, not otherwise specified; T cell lymphoma, NOS, T‐cell lymphoma, not otherwise specified; T‐LGLL, T‐cell large granular lymphocytic leukemia; WM, Waldenstrom macroglobulinemia.

^a^ In our study, we included intravascular large B cell lymphoma (IVLBCL) in large B‐cell lymphoma. Four and two cases with a definite diagnosis of IVLBCL was present in the elderly and young cohorts, respectively.

### 3.3 Treatments and outcomes for elderly patients with lymphoma‐associated HLH

**FIGURE 1:**
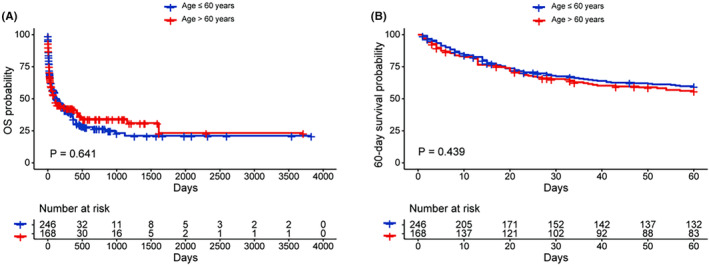
The OS (A) and 60‐day survival probabilities (B) of elderly (age >60 years) and young (age ≤60 years) patients with lymphoma‐associated hemophagocytic lymphohistiocytosis. OS, overall survival.

**FIGURE 2:**
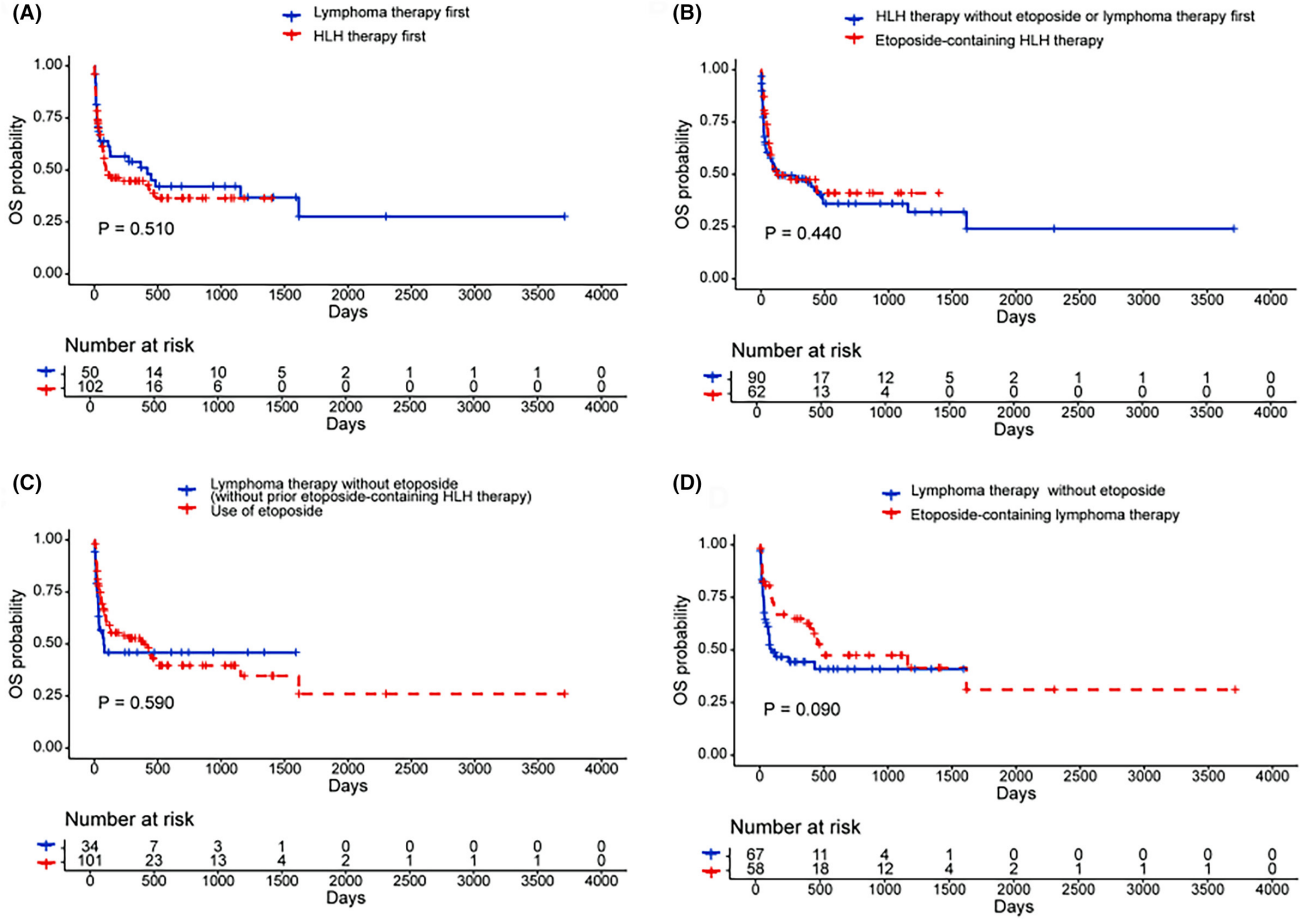
The OS of elderly patients with lymphoma‐associated HLH who received different patterns of treatments (A–D). HLH, hemophagocytic lymphohistiocytosis; OS, overall survival.

A total of 168 elderly patients with LA‐HLH had follow‐up data and the median follow‐up was 54 days. These patients were further analyzed. The treatments for these patients were summarized in Table S1. A total of 102 patients received HLH therapy as the initial treatment. Among patients who received HLH therapy, 62 patients received etoposide‐containing HLH therapy. A total of 125 patients received lymphoma‐directed therapy, and 50 patients received lymphoma‐directed therapy directly without a prior HLH therapy. A total of 101 patients were treated with etoposide, in initial HLH‐directed or lymphoma‐directed therapy. Sixteen patients did not receive either HLH therapy or lymphoma‐directed therapy, mostly due to the rapid progression of the disease. The median survival for elderly patients was 92 days, and the 60‐day survival probability was 56.2%. No significant differences were observed in OS and 60‐day survival probabilities between the elderly and younger patients (median survival: elderly 92 days vs. young 126 days, *p* = 0.641, Figure 1A; 60‐day survival probabilities: elderly 56.2% vs. young 59.9%, *p* = 0.439, Figure 1B). In subgroup analysis based on lymphoma subtypes (B cell lymphoma or T/NK cell lymphoma or Hodgkin lymphoma), the survival outcomes did not differ between the elderly and younger patients (Figure S1). Patients who did not receive either HLH therapy or lymphoma‐directed therapy (*n* = 16) had a very dismal prognosis, with a median survival of only 4 days. Patients treated with HLH therapy and those who received lymphoma‐directed therapy without prior HLH therapy showed similar OS (*p* = 0.510, Figure 2A) and 60‐day survival probabilities (*p* = 0.760, Figure S2A). The use of etoposide‐containing HLH therapy was not associated with improved OS (*p* = 0.440, Figure 2B) or 60‐day survival (*p* = 0.410, Figure S2B). Outcomes of patients who received etoposide treatment in HLH‐directed or lymphoma‐directed therapy were similar to those who received lymphoma‐directed therapy without any etoposide regimen (OS, *p* = 0.590, Figure 2C; 60‐day survival, *p* = 0.160, Figure S2C). In patients who received lymphoma‐directed therapy, the use of etoposide‐containing lymphoma‐directed therapy was associated with improved overall survival, although the difference was not statistically significant (*p* = 0.090, Figure 2D); the use of etoposide‐containing lymphoma‐directed therapy was significantly associated with improved 60‐day survival (*p* = 0.025, Figure S2D).

We then separately analyzed the role of etoposide in B‐cell LA‐HLH and T/NK‐cell LA‐HLH. For B‐cell LA‐HLH, we found that etoposide‐containing HLH therapy was not associated with improved outcomes (OS or 60‐day survival, Figure S3A,D). The use of etoposide was associated with improved 60‐day survival (*p* = 0.004, Figure S3E) but OS (*p* = 0.310, Figure S3B). For B‐cell LA‐HLH, etoposide‐containing lymphoma‐directed therapy showed an advantage over lymphoma‐therapy without etoposide in improving 60‐day survival (*p* = 0.012, Figure S3F) but not OS (*p* = 0.140, Figure S3C). For T/NK‐cell LA‐HLH, etoposide‐containing HLH therapy, etoposide use, or etoposide‐containing lymphoma‐directed therapy was not associated with improved OS or 60‐day survival (Figure S3A–F).

### 3.4 Prognostic factors

**FIGURE 3:**
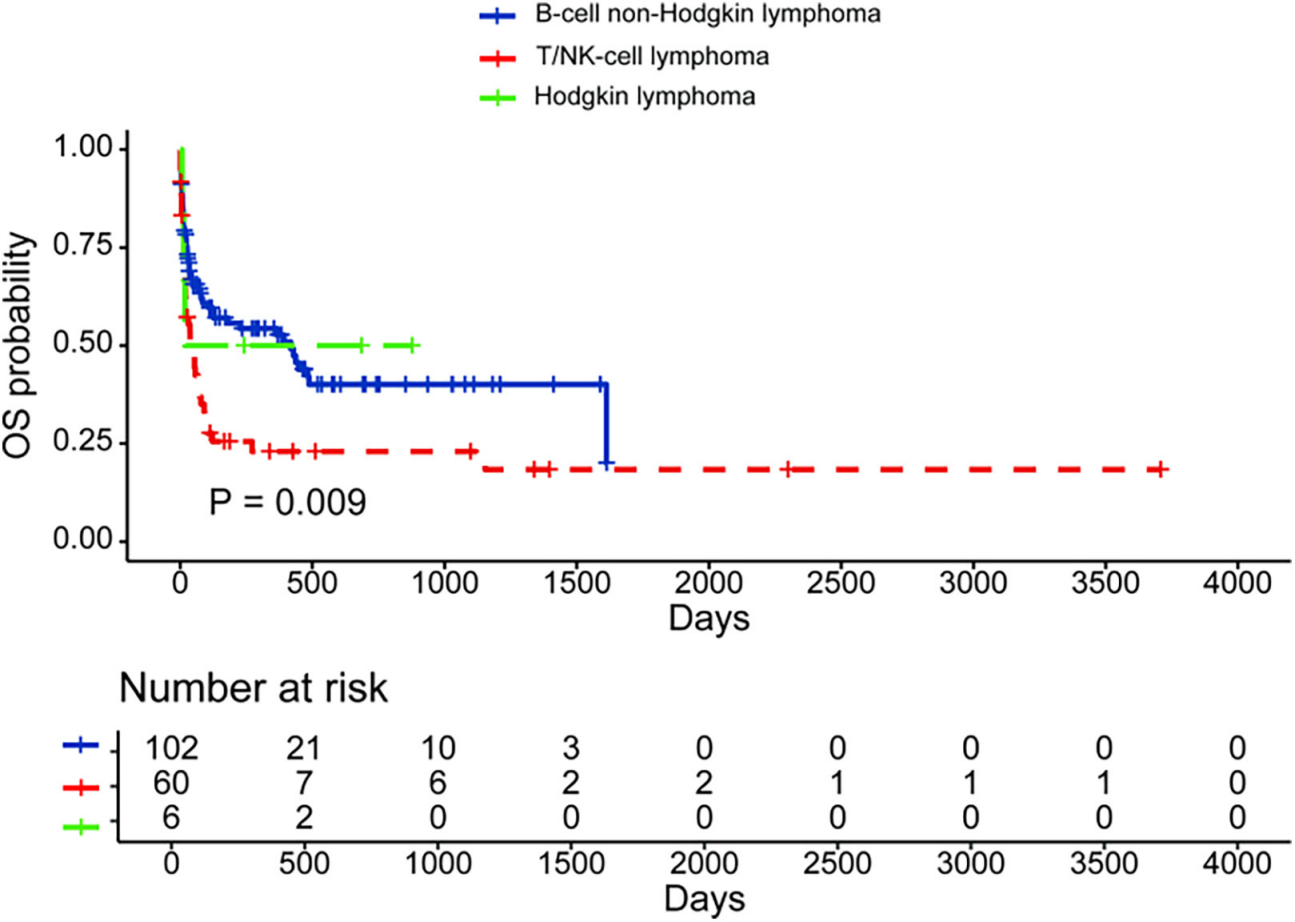
The impact of pathological subtypes on OS of elderly patients with lymphoma‐associated hemophagocytic lymphohistiocytosis. OS, overall survival.

**FIGURE 4:**
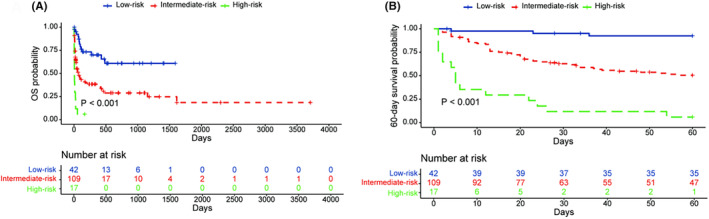
The OS (A) and 60‐day survival (B) in elderly patients with lymphoma‐associated hemophagocytic lymphohistiocytosis according to the prognostic index. OS, overall survival.

We analyzed potential prognostic markers for elderly patients with LA‐HLH. We found that lymphoma subtypes had a significant impact on OS in elderly patients with LA‐HLH (*p* = 0.009, Figure 3). Patients with NK‐cell neoplasm had a median survival of only 21 days. In addition to lymphoma subtypes, we also analyzed the prognostic roles of age, hemoglobin, neutrophil counts, platelets, fibrinogen, LDH, ferritin, albumin, and creatinine (Table 3). The best cutoffs of age, hemoglobin, neutrophil counts, platelets, fibrinogen, LDH, albumin, and creatinine were shown in Table 3. In univariate analysis, the T/NK‐cell subtype, a lower platelet count (≤53 × 10^9^/L), a lower albumin level (≤32.1 g/L), a higher LDH level (>1407 U/L), a higher creatinine level (>96.8 μmol/L), and a lower fibrinogen level (≤2.59 g/L) predicted worse OS. Further multivariate analysis demonstrated that the T/NK‐cell subtype, a lower platelet count, a lower albumin level, a higher LDH level, and a higher creatinine level were independent predictors of decreased OS. The lymphoma subtype, platelets, albumin, LDH, and creatinine were also independent prognostic factors for the 60‐day survival (Table S2). These five factors were used to construct a prognostic index for elderly patients with LA‐HLH. Weighted risk scores of 1 were assigned to each factor and the total risk scores ranged from 0 to 5. Patients were divided into three risk groups: low‐risk (risk score: 0–1), intermediate‐risk (risk score: 2–3), and high‐risk (risk score: 4–5). Patients in different risk groups showed significantly different outcomes. The median survival for patients in the low‐risk, intermediate‐risk, and high‐risk groups were not reached, 74 days, and 5 days, respectively (*p* < 0.001, Figure 4A). The 60‐day survival probabilities for the three groups were also significantly different (high‐risk 92.4% vs. intermediate‐risk 50.4% vs. low‐risk 5.9%, *p* < 0.001, Figure 4B).

**TABLE 3:** Risk factors for overall survival in elderly patients with lymphoma‐associated hemophagocytic lymphohistiocytosis.

	Univariate analysis			Multivariate analysis		
	HR	95% CI	*p* Value	HR	95% CI	*p* Value
Age >77 years	1.73	1.00–3.01	0.052			
T/NK‐cell lymphoma	1.86	1.25–2.77	0.002	2.47	1.59–3.84	<0.001
Hemoglobin ≤102 g/L	1.34	0.73–2.46	0.337			
Platelets ≤53 × 10^9^/L	2.36	1.46–3.79	<0.001	1.92	1.15–3.19	0.012
Neutrophils ≤0.99 × 10^9^/L	1.31	0.80–2.16	0.287			
Albumin ≤32.1 g/L	1.84	1.06–3.20	0.030	1.80	1.02–3.19	0.042
Creatinine >96.8 μmol/L	1.94	1.17–3.20	0.010	1.69	1.01–2.82	0.045
LDH >1407 U/L	2.84	1.81–4.45	<0.001	2.75	1.70–4.44	<0.001
Fibrinogen ≤2.59 g/L	2.11	1.27–3.53	0.004	1.48	0.86–2.54	0.153
Serum ferritin >1500 μg/L	1.27	0.82–1.97	0.283			

## 4 DISCUSSION

In the current study, we retrospectively analyzed a large cohort of elderly patients with LA‐HLH. We found that elderly patients with LA‐HLH showed different clinical characteristics and lymphoma subtypes from young patients. We found that the triggers underlying LA‐HLH in elderly patients were significantly different from those in young patients. B‐cell NHL was more common in elderly patients while T/NK‐cell lymphoma was more common in young patients. Regarding specific subtypes, large B‐cell lymphoma was much more prevalent in elderly patients with LA‐HLH, while ANKL and ENKL were more common in young patients. The prognosis of elderly patients with LA‐HLH was poor, and those with T/NK‐cell subtypes had an extremely poor prognosis. Initial HLH therapy or Initial etoposide‐containing HLH therapy did not seem to the survival of elderly patients with LA‐HLH. Use of etoposide or etoposide‐containing lymphoma‐directed therapy was not associated improved OS of elderly patients with LA‐HLH.

Identification of the triggers for secondary HLH is important for treatment decisions in patients with HLH. Understanding the lymphoma subtypes underlying HLH could be helpful for differential diagnosis in patients with secondary HLH.^3^ According to most of previous studies, T/NK‐cell lymphoma is more common than B‐cell NHL as a trigger for HLH.^3^, ^5^, ^10^ However, these studies did not separately analyze the young and old patients. According to a previous study, the most common subtypes in children or adolescents with malignancy‐associated HLH are anaplastic large cell lymphoma (ALCL) and lymphoblastic lymphoma.^11^ This finding suggests that the distribution of lymphoma subtypes may vary in different age groups. Our study revealed that the distribution of lymphoma subtypes was different between different age groups in adult patients with LA‐HLH. This could be attributed to the impacts of age on the incidences of specific subtypes of lymphoid malignancies. For instance, ANKL patients, who frequently present with HLH, are mostly young to middle‐aged adults.^12^ Intravascular large B‐cell lymphoma (IVLBCL), another subtype that is associated with HLH, is more common in the elderly population.^13^ However, as the distribution of lymphoma subtypes is different in different ethnic populations, for example, ANKL and ENKL is much more prevalent among Asians than in other populations,^14^, ^15^ our findings regarding the lymphoma subtypes may not apply to other populations.

The prognosis of elderly patients with LA‐HLH is poor, with a median survival of only 92 days. The difference in outcomes between elderly and young patients is not significant and this finding is observed in different subgroups (B‐cell lymphoma or T/NK‐cell lymphoma or Hodgkin lymphoma). The underlying reason for this phenomenon needs to be explored. The treatment for elderly patients with LA‐HLH is challenging, as elderly lymphoma patients have more comorbidities and reduced tolerance to chemotherapies. The current treatment recommendation for LA‐HLH is using HLH‐directed therapy containing etoposide followed by lymphoma‐directed therapy, which is also called a two‐step approach.^16^ However, the role of HLH‐directed therapy including the HLH‐94 regimen in the treatment of LA‐HLH remains controversial, primarily due to the lack of prospective randomized trials. A possible concern for the use of lymphoma‐directed therapy is the intolerance of patients with HLH, as these patients always have poor performance status, severe cytopenia, and poor organ functions. The direct use of anti‐lymphoma therapy may lead to increased therapy‐related mortalities. Previous studies suggested, that the use of etoposide, no matter in the HLH‐directed or lymphoma‐directed strategy, was associated with better outcomes.^17^, ^18^ Our study did not show the use of HLH therapy or etoposide‐containing HLH therapy was associated with improved outcomes. Additionally, the use of etoposide or etoposide‐containing lymphoma therapy did not improve the OS of elderly patients with LA‐HLH. These findings were not consistent with those from previous studies. However, in our study, for patients who received lymphoma‐directed therapy, the use of etoposide‐containing chemotherapy was associated with better 60‐day survival. Interestingly, for elderly patients with B‐cell LA‐HLH, the use of etoposide or etoposide‐containing lymphoma therapy was significantly associated with better 60‐day survival but not OS. How to translate better 60‐day survival into better OS needs to be explored in the future. Despite these findings, it should be mentioned that the selection of treatments for elderly patients with LA‐HLH as well as etoposide doses need to be dependent on the organ functions and general conditions of these patients.

The poor prognosis of elderly patients with LA‐HLH emphasizes an unmet need for therapeutic options for this population. Although there is ongoing debate over the treatment strategy for patients with LA‐HLH, we think that effective lymphoma‐directed therapy is critical for improving the survival of patients with LA‐HLH, especially long‐term survival. The advent of novel agents may improve the outcomes of specific subgroups of elderly patients with LA‐HLH. For example, most of cases of IVLBCL, which is frequently associated with HLH,^19^, ^20^, ^21^ are primarily MCD‐type large B‐cell lymphoma.^22^ As patients with MCD‐type DLBCL may benefit from the addition of Bruton's tyrosine kinase inhibitors,^23^ the use of Bruton's tyrosine kinase inhibitors could be promising in treating patients with IVLBCL‐associated HLH. According to a phase 2 trial, all but one patient with IVLBCL who received zanubrutinib plus R‐CHOP achieved complete response at the end of the therapy and no relapses occurred, suggesting this regimen is highly effective in patients with IVLBCL.^24^ Elderly patients with T/NK‐cell LA‐HLH had an even worse outcome and those with NK‐cell lymphoid malignancy had a median overall survival of only 21 days. The anti‐PD‐1 antibody or anti‐PD‐L1 antibody has been demonstrated to be effective in treating ENKL.^25^, ^26^, ^27^ According to a retrospective study by He et al, the use of a combination of anti‐PD‐1 antibody plus chemotherapy seemed to be associated with prolonged survival in patients with ENKL‐associated HLH.^28^ The efficacy and safety of the regimens containing anti‐PD‐1 or anti‐PD‐L1 antibodies in elderly patients with ENKL‐associated HLH need to be confirmed in future prospective trials. Novel agents including JAK2 inhibitor ruxolitinib and the anti–IFN‐γ monoclonal antibody emapalumab have shown promising effects in children with primary HLH.^29^, ^30^ These agents may have a role in the treatment of elderly patients with LA‐HLH, especially for those who do not tolerate routine therapy.

We analyzed the prognostic factors for elderly patients with LA‐HLH. T/NK‐cell subtype, lower platelet count, lower albumin level, higher LDH level, and higher creatinine were identified as independent factors for predicting poor prognosis for elderly patients with LA‐HLH. The T/NK‐cell subtype has been demonstrated as a negative predictor of survival in patients with LA‐HLH,^5^ as cases of the T/NK‐cell type usually show a highly aggressive clinical course. Low platelet and increased creatinine have also been demonstrated as prognostic factors in patients with HLH.^31^, ^32^ Low platelet counts and increased creatinine are associated with disease severity and indicate decreased tolerance to conventional therapy. By establishing a prognostic index comprising five factors, we categorized elderly patients into three groups and identified a group of patients at a very high risk of early death. The median survival for this group of patients was only 5 days. They are unlikely to tolerate routine therapies and novel treatments including emapalumab may be of potential efficacy in these patients.

Our study has limitations due to its retrospective nature. A precise diagnosis was not available for some cases in this cohort. For example, some cases could only be diagnosed as B‐cell non‐Hodgkin lymphoma or T cell lymphoma, not otherwise specified. The pathology of lymphoma was not centrally reviewed in this study. The baseline characteristics including performance status could affect the selection of treatments and could be confounding factors in analyzing the prognostic impacts of different treatments. However, this was not analyzed in the current study. Moreover, the patterns of treatment failures were not available. Prospective studies are warranted to address these limitations.

In conclusion, our study demonstrated that elderly patients with LA‐HLH displayed different clinical characteristics and lymphoma subtypes from young patients. The prognosis of elderly patients with LA‐HLH is poor, especially for those with T/NK‐cell lymphoid malignancies, suggesting an unmet need in the treatment of elderly patients with LA‐HLH. The initial use of HLH therapy or etoposide‐containing HLH therapy was not associated with improved OS. Additionally, the use of etoposide, in HLH‐directed or lymphoma‐directed therapy, was not associated with improved OS. A five‐point prognostic index could categorize elderly patients with LA‐HLH into three groups with different survival outcomes. Future clinical trials are warranted to explore better therapeutic approaches for elderly patients with LA‐HLH.

## AUTHOR CONTRIBUTIONS

**Yi Miao:** Conceptualization (equal); funding acquisition (equal); methodology (equal); supervision (equal). **Jing Zhang:** Data curation (equal); formal analysis (equal); methodology (equal); visualization (equal); writing – original draft (equal). **Xuzhang Lu:** Data curation (equal); formal analysis (equal); methodology (equal); visualization (equal); writing – original draft (equal). **Meng Wu:** Data curation (equal); formal analysis (equal); methodology (equal); visualization (equal); writing – original draft (equal). **Bingzong Li:** Data curation (equal); writing – original draft (equal). **Liang Yu:** Data curation (equal); writing – original draft (equal). **Miao Sun:** Data curation (equal); writing – original draft (equal). **Yun Zhuang:** Data curation (equal); writing – original draft (equal). **Yuqing Miao:** Data curation (equal); writing – original draft (equal). **Haiwen Ni:** Data curation (equal); writing – original draft (equal). **Xiaoyan Xie:** Data curation (equal); writing – original draft (equal). **Jingyan Xu:** Data curation (equal); writing – original draft (equal). **Yunping Zhang:** Data curation (equal); writing – original draft (equal). **Min Zhao:** Data curation (equal); writing – original draft (equal). **Min Xu:** Data curation (equal); writing – original draft (equal). **Wanchuan Zhuang:** Data curation (equal); writing – original draft (equal). **Weiying Gu:** Data curation (equal); writing – original draft (equal). **Guoqiang Lin:** Data curation (equal); writing – original draft (equal). **Haiying Hua:** Data curation (equal); writing – original draft (equal). **Jianfeng Zhu:** Data curation (equal); writing – original draft (equal). **Maozhong Xu:** Data curation (equal); writing – original draft (equal). **Tao Jia:** Data curation (equal); writing – original draft (equal). **Ping Liu:** Data curation (equal); writing – original draft (equal). **Lijia Zhai:** Data curation (equal); writing – original draft (equal). **Tongtong Zhang:** Data curation (equal); writing – original draft (equal). **Qiurong Shan:** Data curation (equal); writing – original draft (equal). **Qiudan Shen:** Data curation (equal); writing – original draft (equal). **Jun Qian:** Data curation (equal); writing – original draft (equal). **Chunling Wang:** Data curation (equal); writing – original draft (equal). **Jianyong Li:** Conceptualization (equal); funding acquisition (equal); methodology (equal); supervision (equal). **Wenyu Shi:** Conceptualization (equal); funding acquisition (equal); methodology (equal); supervision (equal).

## FUNDING INFORMATION

This work was supported by the Nature Science Foundation of Jiangsu Province (BK20210962), the Social Development Project of Jiangsu Province Science and Technology Plan (BE2023775), Jiangsu Province Capability Improvement Project through Science, Technology and Education (ZDXK202209), and Postgraduate Research & Practice Innovation Program of Jiangsu Province (JX10214021).

## CONFLICT OF INTEREST STATEMENT

None.

## ETHICS STATEMENT

This study was approved by the Ethics Committee of the Affiliated Hospital of Nantong University (approval number: 2023‐K052‐01). The study was conducted according to the 1964 Helsinki Declaration and its later amendments.

## PATIENT CONSENT STATEMENT

Patient consent was waived because this was a retrospective and deidentified study.

## Supporting information


Appendix S1.

## Data Availability

The data that support the findings of this study are available on request from the corresponding author.

## References

[1] Canna SW , Marsh RA . Pediatric hemophagocytic lymphohistiocytosis. Blood. 2020;135(16):1332‐1343. 32107531 10.1182/blood.2019000936 PMC8212354

[2] Ramos‐Casals M , Brito‐Zeron P , Lopez‐Guillermo A , Khamashta MA , Bosch X . Adult haemophagocytic syndrome. Lancet. 2014;383(9927):1503‐1516. 24290661 10.1016/S0140-6736(13)61048-X

[3] Miao Y , Zhang J , Chen Q , et al. Spectrum and trigger identification of hemophagocytic lymphohistiocytosis in adults: a single‐center analysis of 555 cases. Front Immunol. 2022;13:970183. 36032133 10.3389/fimmu.2022.970183 PMC9411524

[4] Zou H , He L , Hue Z , et al. Serum sCD25/ferritin ratio combined with MCP‐1 is a valid predictor for identifying LAHS with HLH as the first manifestation. J Cancer Res Clin Oncol. 2023;149(11):8521‐8533. 37093345 10.1007/s00432-023-04781-4 PMC11798138

[5] Zhao AL , Li M , Li LF , et al. Clinical characteristics and prognosis of lymphoma‐associated hemophagocytic syndrome. Zhonghua Yi Xue Za Zhi. 2022;102(28):2173‐2180. 35872581 10.3760/cma.j.cn112137-20220221-00349

[6] Knauft J , Schenk T , Ernst T , et al. Lymphoma‐associated hemophagocytic lymphohistiocytosis (LA‐HLH): a scoping review unveils clinical and diagnostic patterns of a lymphoma subgroup with poor prognosis. Leukemia. 2024;38(2):235‐249. 38238443 10.1038/s41375-024-02135-8 PMC10844097

[7] Birndt S , Schenk T , Heinevetter B , et al. Hemophagocytic lymphohistiocytosis in adults: collaborative analysis of 137 cases of a nationwide German registry. J Cancer Res Clin Oncol. 2020;146(4):1065‐1077. 32076823 10.1007/s00432-020-03139-4 PMC7085479

[8] Setiadi A , Zoref‐Lorenz A , Lee CY , Jordan MB , Chen LYC . Malignancy‐associated haemophagocytic lymphohistiocytosis. Lancet Haematol. 2022;9(3):e217‐e227. 35101205 10.1016/S2352-3026(21)00366-5

[9] Henter JI , Horne A , Arico M , et al. HLH‐2004: diagnostic and therapeutic guidelines for hemophagocytic lymphohistiocytosis. Pediatr Blood Cancer. 2007;48(2):124‐131. 16937360 10.1002/pbc.21039

[10] Zhang Q , Li L , Zhu L , et al. Adult onset haemophagocytic lymphohistiocytosis prognosis is affected by underlying disease: analysis of a single‐institution series of 174 patients. Swiss Med Wkly. 2018;148:w14641. 30378643 10.4414/smw.2018.14641

[11] Lehmberg K , Sprekels B , Nichols KE , et al. Malignancy‐associated haemophagocytic lymphohistiocytosis in children and adolescents. Br J Haematol. 2015;170(4):539‐549. 25940575 10.1111/bjh.13462

[12] El Hussein S , Medeiros LJ , Khoury JD . Aggressive NK cell leukemia: current state of the art. Cancers (Basel). 2020;12(10):2900. 33050313 10.3390/cancers12102900 PMC7600035

[13] Shimada K , Kinoshita T , Naoe T , Nakamura S . Presentation and management of intravascular large B‐cell lymphoma. Lancet Oncol. 2009;10(9):895‐902. 19717091 10.1016/S1470-2045(09)70140-8

[14] Kommalapati A , Tella SH , Ganti AK , Armitage JO . Natural killer/T‐cell neoplasms: analysis of incidence, patient characteristics, and survival outcomes in the United States. Clin Lymphoma Myeloma Leuk. 2018;18(7):475‐479. 29752210 10.1016/j.clml.2018.04.009

[15] Adams SV , Newcomb PA , Shustov AR . Racial patterns of peripheral T‐cell lymphoma incidence and survival in the United States. J Clin Oncol. 2016;34(9):963‐971. 26962200 10.1200/JCO.2015.63.5540 PMC5070555

[16] Lee JC , Logan AC . Diagnosis and management of adult malignancy‐associated hemophagocytic lymphohistiocytosis. Cancers (Basel). 2023;15(6):1839. 36980725 10.3390/cancers15061839 PMC10046521

[17] Song Y , Wang J , Wang Y , Wu L , Wang Z . Requirement for containing etoposide in the initial treatment of lymphoma associated hemophagocytic lymphohistiocytosis. Cancer Biol Ther. 2021;22(10–12):598‐606. 34724875 10.1080/15384047.2021.1996139 PMC8726658

[18] Lee CY , Wills B , Vardhana SA , Moskowitz AJ . Clinical characteristics and outcomes of adult lymphoma‐associated hemophagocytic lymphohistiocytosis (HLH). J Clin Oncol. 2021;39(15_suppl):e19526.

[19] Zhang Y , Zhu TN , Sun J , Zhong DR , Zhang W , Zhou DB . Clinical characteristics of intravascular large B cell lymphoma: a single‐center retrospective study. Zhonghua Xue Ye Xue Za Zhi. 2018;39(12):1004‐1009. 30612402 10.3760/cma.j.issn.0253-2727.2018.12.007 PMC7348217

[20] Murase T , Nakamura S . An Asian variant of intravascular lymphomatosis: an updated review of malignant histiocytosis‐like B‐cell lymphoma. Leuk Lymphoma. 1999;33(5–6):459‐473. 10342574 10.3109/10428199909058451

[21] Matsue K , Abe Y , Narita K , et al. Diagnosis of intravascular large B cell lymphoma: novel insights into clinicopathological features from 42 patients at a single institution over 20 years. Br J Haematol. 2019;187(3):328‐336. 31267524 10.1111/bjh.16081 PMC6900202

[22] Shimada K , Yoshida K , Suzuki Y , et al. Frequent genetic alterations in immune checkpoint‐related genes in intravascular large B‐cell lymphoma. Blood. 2021;137(11):1491‐1502. 33512416 10.1182/blood.2020007245 PMC7976508

[23] Wilson WH , Wright GW , Huang DW , et al. Effect of ibrutinib with R‐CHOP chemotherapy in genetic subtypes of DLBCL. Cancer Cell. 2021;39(12):1643‐1653.e3. 34739844 10.1016/j.ccell.2021.10.006 PMC8722194

[24] Zhang Y , Chen C , Zhao D , et al. A prospective single‐center phase 2 study of zanubrutinib plus R‐CHOP in treat‐naïve intravascular large B cell lymphoma. HemaSphere. 2023;7(S3):e1662573.

[25] Tao R , Fan L , Song Y , et al. Sintilimab for relapsed/refractory extranodal NK/T cell lymphoma: a multicenter, single‐arm, phase 2 trial (ORIENT‐4). Signal Transduct Target Ther. 2021;6(1):365. 34702811 10.1038/s41392-021-00768-0 PMC8548511

[26] Kim SJ , Lim JQ , Laurensia Y , et al. Avelumab for the treatment of relapsed or refractory extranodal NK/T‐cell lymphoma: an open‐label phase 2 study. Blood. 2020;136(24):2754‐2763. 32766875 10.1182/blood.2020007247

[27] Huang H , Tao R , Hao S , et al. Sugemalimab monotherapy for patients with relapsed or refractory extranodal natural killer/T‐cell lymphoma (GEMSTONE‐201): results from a single‐arm, multicenter, phase II study. J Clin Oncol. 2023;41(16):3032‐3041. 36996373 10.1200/JCO.22.02367 PMC10414714

[28] He Y , Gao Y , Ping L , et al. The emerging role of anti‐PD‐1 antibody‐based regimens in the treatment of extranodal NK/T‐cell lymphoma‐associated hemophagocytic lymphohistiocytosis. J Cancer Res Clin Oncol. 2023;149(5):2017‐2027. 35809114 10.1007/s00432-022-04147-2 PMC11797699

[29] Ge J , Zhang Q , Ma H , et al. Ruxolitinib‐based regimen in children with primary hemophagocytic lymphohistiocytosis. Haematologica. 2023;109:458‐465. 10.3324/haematol.2023.283478 PMC10828753 37470145

[30] Locatelli F , Jordan MB , Allen C , et al. Emapalumab in children with primary hemophagocytic Lymphohistiocytosis. N Engl J Med. 2020;382(19):1811‐1822. 32374962 10.1056/NEJMoa1911326

[31] Zhang J , Qin S , Jin Z , et al. The clinical significance and prognostic role of whole‐blood Epstein‐Barr virus DNA in lymphoma‐associated hemophagocytic lymphohistiocytosis. J Clin Immunol. 2023;43(6):1302‐1310. 37093406 10.1007/s10875-023-01493-9

[32] Wang D , Tong X , Liu S , et al. Clinical characteristics and risk factors for 90‐day overall survival among 204 adult patients with secondary hemophagocytic lymphohistiocytosis: experience from a single‐center retrospective study. Front Med (Lausanne). 2022;9:774959. 36300188 10.3389/fmed.2022.774959 PMC9589347

