# Supplementary material for: Clinicopathological characteristics, prognostic factors, and outcomes of elderly patients with lymphoma‐associated hemophagocytic lymphohistiocytosis: A multicenter analysis
Source: Cancer Med. 2024 Sep 1;13(16):e70178. doi: 10.1002/cam4.70178 (PMC11366774; doi:10.1002/cam4.70178)

**Supplementary Table 1. HLH and lymphoma-directed regimens in elderly patients with LA-HLH**

|  | **Total** | **B-cell NHL** | **T/NK-cell NHL** | **HL** |
| --- | --- | --- | --- | --- |
| **Number of patients^*^** | 168 | 102 | 60 | 6 |
| **No HLH or lymphoma-directed therapy** | 16 (9.5) | 7 (6.9) | 8 (13.3) | 1 (16.7) |
| **HLH-directed therapy** | 102 (60.7) | 61 (59.8) | 38 (63.3) | 3 (50) |
| Corticosteroids only | 34 (20.2) | 16 (15.7) | 17 (28.3) | 1 (16.7) |
| Etoposide-containing regimens | 62 (36.9) | 41 (40.2) | 19 (31.7) | 2 (33.3) |
| HLH-94/modified HLH-94 protocols | 41 (24.4) | 29 (28.4) | 11 (18.3) | 1 (16.7) |
| HLH-2004 protocol | 3 (1.8) | 1 (1.0) | 2 (3.3) | 0 |
| DEP or DEP-like regimen | 13^#^ (7.7) | 9 (8.8) | 3 (5) | 1 (16.7) |
| Etoposide alone | 4 (2.4) | 2 (2.0) | 2 (3.3) | 0 |
| Other etoposide-containing regimens | 2 (1.2) | 1 (1.0) | 1 (1.7) | 0 |
| IVIG alone or IVIG plus corticosteroids | 5 (3.0) | 3 (2.9) | 2 (3.3) | 0 |
| Cyclophosphamide alone | 1 (0.6) | 1 (1.0) | 0 | 0 |
| Use of ruxolitinib | 2 (1.2) | 2 (2.0) | 0 | 0 |
| **Lymphoma-directed therapy^##^** | 125 (74.4) | 82 (80.4) | 39 (65) | 4 (66.7) |
| Rituximab-containing regimen | 74 (44.0) | 73 (71.6) | 1 (1.7) | 0 |
| CHOP | 32 (19.0) | 25 (24.5) | 5 (8.3) | 2 (33.3) |
| CHOPE | 19 (11.3) | 14 (13.7) | 5 (8.3) | 0 |
| DA-EPOCH | 16 (9.5) | 11 (10.8) | 5 (8.3) | 0 |
| CEOP | 12 (7.1) | 8 (7.8) | 4 (6.7) | 0 |
| GemOx | 8 (4.8) | 5 (4.9) | 3 (5) | 0 |
| MINE | 6 (3.6) | 4 (3.9) | 2 (3.3) | 0 |
| DEP | 4 (2.4) | 3 (2.9) | 1 (1.7) | 0 |
| Asparaginase-containing regimen | 7 (4.2) | 0 | 7 (11.7) | 0 |
| PMED | 4 (2.4) | 0 | 4 (6.7) | 0 |
| PD-1 antibody containing regimen | 15 (8.9) | 5 (4.9) | 8 (13.3) | 2 (33.3) |
| Low-intensity therapies only^###^ | 17 (10.1) | 10 (9.8) | 7 (11.7) | 0 |
| More than one therapies | 18 (10.7) | 11 (10.8) | 6 (10) | 1 (16.7) |

* Treatment details of follow-up data were shown here.

# One patient receiving DEP regimen following use of the HLH-94 protocol.

## lymphoma regimens after failure with the initial lymphoma therapy were also included, and stem cell transplantation was not included.

###Low-intensity therapies indicate monoclonal antibodies or oral agents.

NHL, non-Hodgkin lymphoma; HL, Hodgkin lymphoma; DEP, liposomal doxorubicin, etoposide and high-dose methylprednisolone; IVIG, intravenous immunoglobulin; CHOP, cyclophosphamide, doxorubicin, vincristine, and prednisolone; CHOPE, CHOP plus etoposide; DA-EPOCH, dose-adjusted etoposide, prednisone, vincristine, cyclophosphamide, and doxorubicin; CEOP, cyclophosphamide, etoposide, vincristine, and prednisolone; GemOx, gemcitabine in combination with oxaliplatin; MINE, mesna, ifosfamide, mitoxantrone, and etoposide; PMED, pegaspargase, etoposide, methotrexate and dexamethasone.

Results are shown as number of patients and corresponding percentages.

**Supplementary Table 2. Risk factors for 60-day survival in elderly patients with lymphoma-associated** **hemophagocytic lymphohistiocytosis**

|  | Univariate analysis | | | Multivariate analysis | | |  |  | | |  | | |
| --- | --- | --- | --- | --- | --- | --- | --- | --- | --- | --- | --- | --- | --- |
|  | HR | 95% CI | P value | HR | 95% CI | P value |  |  |  |  |  |  |  |
| Age > 77 years | 1.70 | 0.91 - 3.17 | 0.094 |  |  |  |  |  |  |  |  |  |  |
| T/NK-cell lymphoma | 1.91 | 1.20 - 3.04 | **0.007** | 2.57 | 1.55 - 4.26 | **<0.001** |  |  |  |  |  |  |  |
| Hemoglobin ≤ 102 g/L | 1.39 | 0.67 - 2.90 | 0.382 |  |  |  |  |  |  |  |  |  |  |
| Platelets ≤ 53×10^9^/L | 3.25 | 1.71 - 6.18 | **<0.001** | 2.37 | 1.22 - 4.62 | **0.011** |  |  |  |  |  |  |  |
| Neutrophils ≤ 0.99×10^9^/L | 1.86 | 1.09 - 3.18 | **0.023** | 1.31 | 0.75 - 2.30 | 0.348 |  |  |  |  |  |  |  |
| Albumin ≤ 32.1 g/L | 2.53 | 1.21 - 5.29 | **0.013** | 2.25 | 1.06 - 4.80 | **0.035** |  |  |  |  |  |  |  |
| Creatinine > 96.8 μmol/L | 2.27 | 1.30 - 3.96 | **0.004** | 1.91 | 1.06 - 3.43 | **0.030** |  |  |  |  |  |  |  |
| LDH > 1407 U/L | 3.28 | 2.00 - 5.40 | **<0.001** | 3.00 | 1.77 - 5.08 | **<0.001** |  |  |  |  |  |  |  |
| Fibrinogen ≤ 2.59 g/L | 2.06 | 1.11 - 3.84 | **0.022** | 1.33 | 0.69 - 2.57 | 0.397 |  |  |  |  |  |  |  |
| Serum ferritin > 1500 μg/L | 1.15 | 0.69 - 1.92 | 0.593 |  |  |  |  |  |  |  |  |  |  |

Factors with p values<0.05 in univariate analysis were included into multivariate analysis. LDH, lactate dehydrogenase.

**Supplementary Figure 1. The overall survival (A) and 60-day survival (B) of elderly patients (age > 60 years) compared to young patients (age ≤ 60 years) with lymphoma-associated HLH stratified by different subtypes.** HLH, hemophagocytic lymphohistiocytosis.


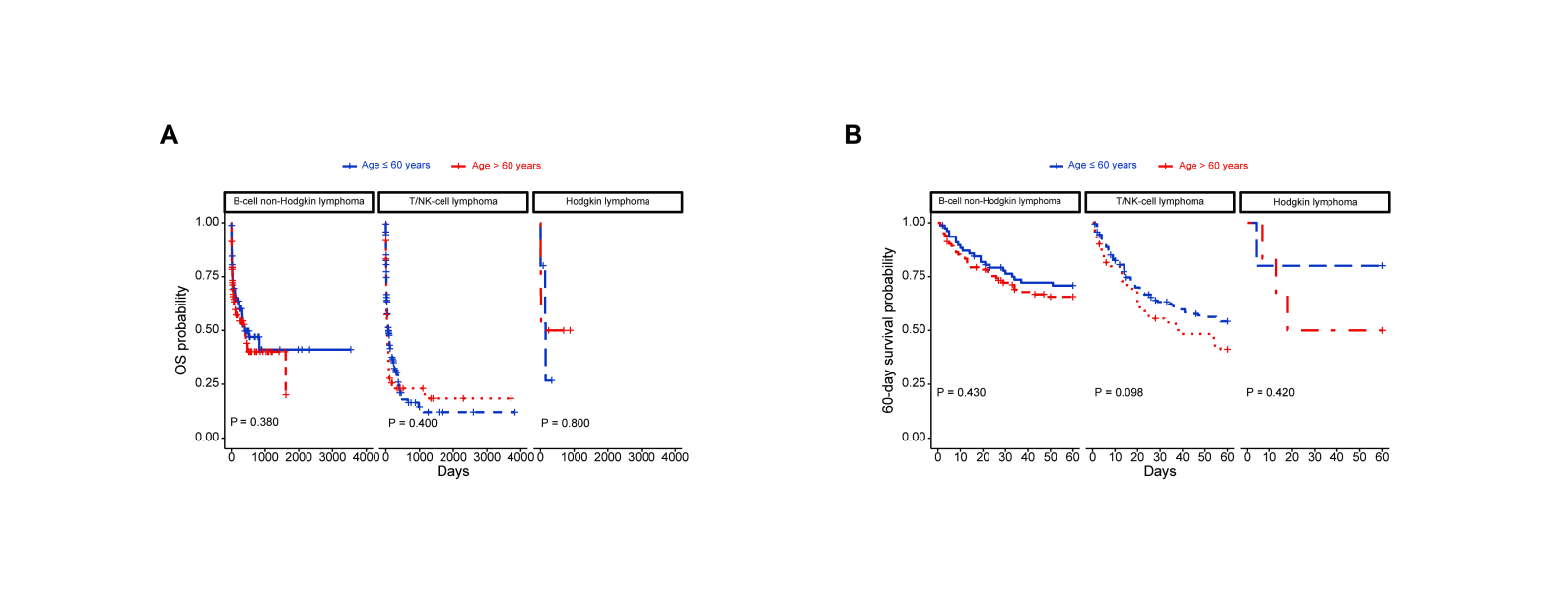


**Supplementary Figure 2. The 60-day survival of elderly patients with lymphoma-associated HLH who received different patterns of treatments (A-D).** HLH, hemophagocytic lymphohistiocytosis.


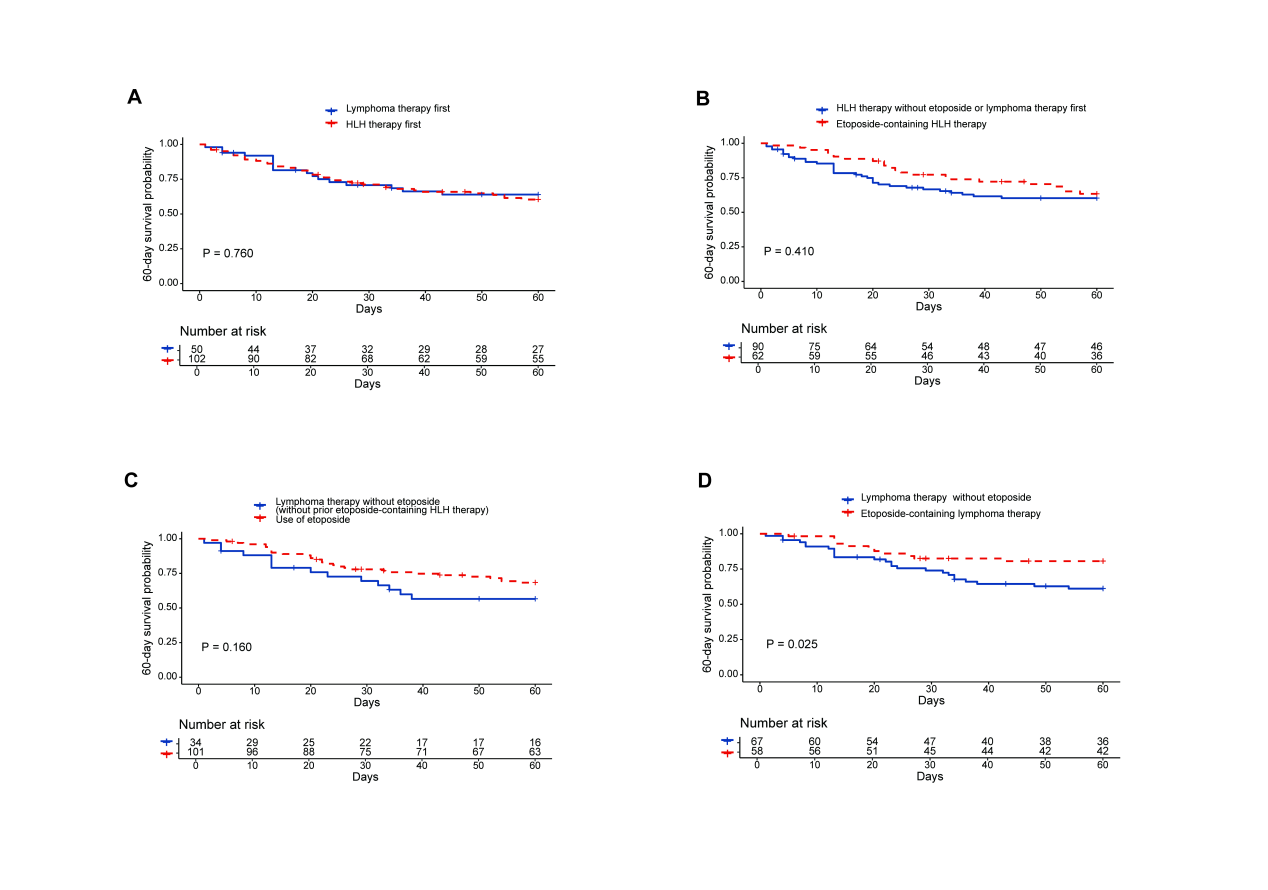


**Supplementary Figure 3. The overall survival (A, B, C) and 60-day survival (D, E, F) of elderly patients with lymphoma-associated HLH who received different patterns of treatments stratified by subtypes.** HLH, hemophagocytic lymphohistiocytosis.


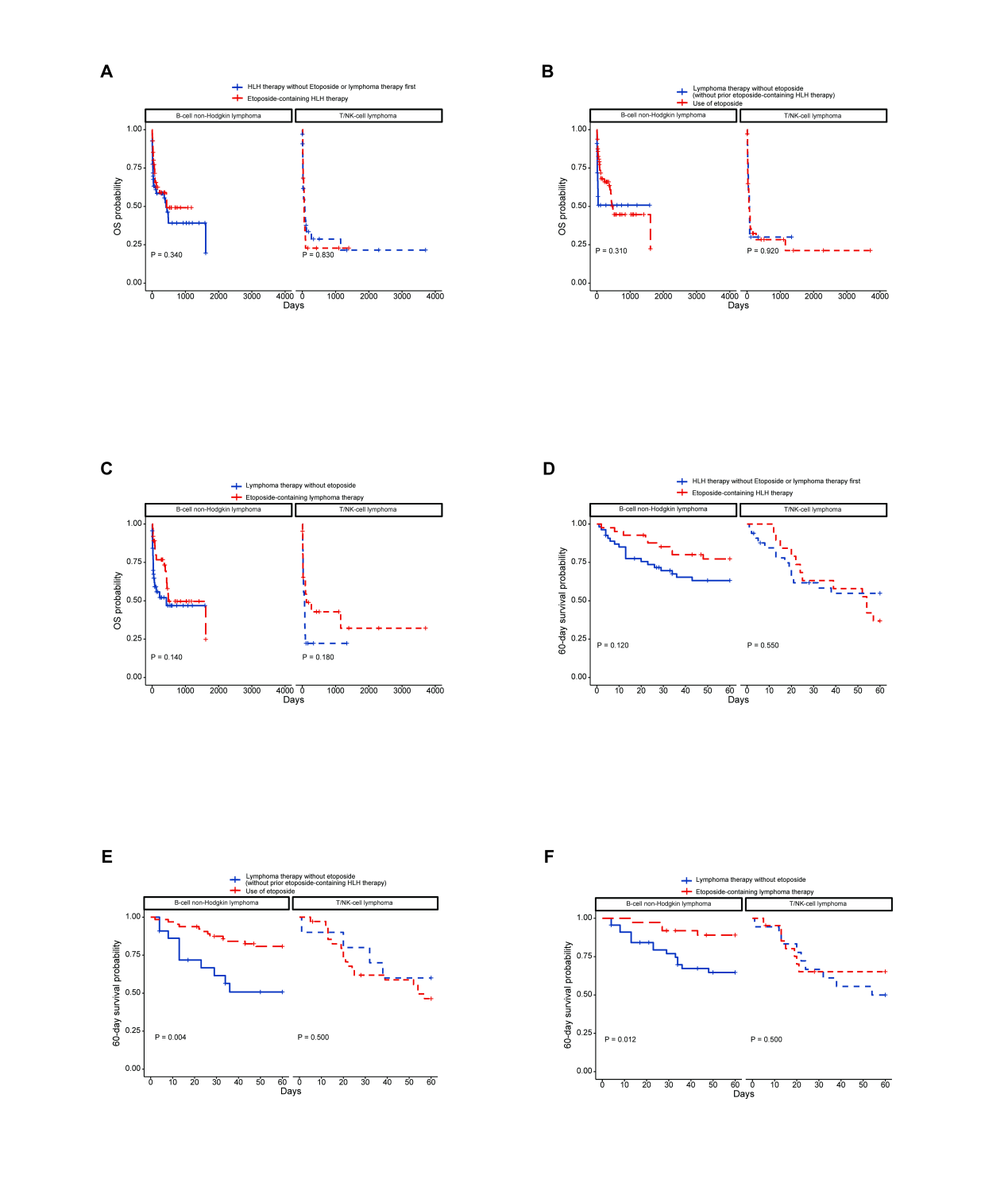

Supplement: Supplementary file 1 — Appendix S1. [file CAM4-13-e70178-s001.docx]
